# Efficient Biodiesel Production Catalyzed by Nanobioconjugate of Lipase from *Pseudomonas fluorescens*

**DOI:** 10.3390/molecules25030651

**Published:** 2020-02-03

**Authors:** Judith-Hajnal Bartha-Vári, Mădălina Elena Moisă, László Csaba Bencze, Florin-Dan Irimie, Csaba Paizs, Monica Ioana Toșa

**Affiliations:** Babeș-Bolyai University, Biocatalysis and Biotransformation Research Center, Arany János 11, 400028 Cluj-Napoca, Romania; varihajni@chem.ubbcluj.ro (J.-H.B.-V.); mmoisa@chem.ubbcluj.ro (M.E.M.); cslbencze@chem.ubbcluj.ro (L.C.B.); irimie@chem.ubbcluj.ro (F.-D.I.); paizs@chem.ubbcluj.ro (C.P.)

**Keywords:** lipase from *Pseudomonas fluorescens*, single-walled carbon nanotubes, biodiesel production

## Abstract

The Amano lipase from *Pseudomonas fluorescens* (L-AK) was covalently immobilized on various carbon nanomaterials (functionalized single-walled carbon nanotubes and graphene oxide) and tested for biodiesel production. Using the most active lipase preparation (covalently immobilized L-AK on SwCNT_NH2_ derivatized with glycerol diglycidyl ether) under optimal conditions, quasi-complete conversion (>99%) of sunflower oil was obtained after only 4 h reaction time. Moreover, the biocatalyst maintained more than 99% of its initial activity in the batch system after multiple recycling experiments.

## 1. Introduction

According to the current consumption, the supply for the most commonly used fossil fuels will last no longer than 50 years [1]. Moreover, the use of these fuels continuously arises pollution and global warming-related problems due to the emitted greenhouse gases. Biodiesel, a mixture of fatty acid alkyl esters, can be an ecofriendly alternative to fossil fuels since it can be obtained from vegetable oils or animal fat. Biodiesel is also biodegradable; it has low toxicity and minimal emissions of sulfates and aromatic compounds [2]. The major feedstock for biodiesel production represents rapeseed oil, followed by soybean and palm oil [3], but also non-edible oils [4], used cooking oils [5], algae [6,7] or fungi oils can also be processed through transesterification with short-chain alcohols for biodiesel production. The most commonly used alcohols for the transesterification reaction are methanol and ethanol. Despite the lower costs of methanol, ethanol remained more preferred due to its low toxicity, biodegradability and the possibility to obtain it through fermentation of renewable resources [8].

Chemical processes using alkali or basic catalysts [9] at high conversion rates of the triglycerides into methyl esters present several drawbacks, including the difficulty of the catalyst removal, the glycerol recovery or the subsequent alkaline/acidic water treatment [10]. Lipase-catalyzed routes represent an environmentally friendly and cleaner alternative having as advantage a smaller number of operational steps [11]. Lipase-catalyzed transesterification processes for biodiesel synthesis using different lipases (lipases from *Candida antarctica*, *Chromobacterium viscosum*, *Mucor miehei*, *Pseudomonas cepacia*, *Pseudomonas fluorescens*, *Porcine pancreatic*, *Rhizomucor miehei* or *Thermomyces lanuginosus*) have been described [12,13,14]. Generally, lipases are inactivated by frequently used short-chain alcohols, such as ethanol or methanol. However, it was demonstrated that Amano lipase from *Pseudomonas fluorescens* has high tolerance toward ethanol and methanol, which makes it suitable for triglycerides transesterification [15,16]. It is also known that free enzymes have a fragile active structure and difficulties concerning their separation and reuse hamper their application in industrial processes [17]. The immobilization of enzymes, resulting in reusable biocatalysts with long-term stability [18,19,20] can overcome these drawbacks. Several immobilization methods have been employed for lipases used for biodiesel synthesis, such as physical adsorption, covalent attachment, entrapment or the use of cross-linked enzyme aggregates [21,22,23].

Covalent immobilization techniques prevent the gradual leak of the enzyme from support, increasing the recyclability and operational stability of the biocatalyst; however, the conformational flexibility of the enzyme, hence the activity, might be affected. Several materials, such as silica, ceramics, polymer resins and magnetic nanoparticles are used as support material for lipase immobilization [24,25,26]. Carbon nanotubes are gaining attention as support material for the immobilization of biomacromolecules, exploiting their electrical, mechanical and thermal properties, and general biocompatibility [27,28]. Moreover, they have the advantage to be strongly hydrophobic, thus keeping the formed glycerol away from the catalytic site of the enzymes. Graphene oxide (GO) displays two accessible sides, thus presents a larger (2630 m^2^ g^−1^) specific surface area compared to carbon nanotubes (1315 m^2^ g^−1^) [29]. Moreover, GO with a high abundance of oxygen-containing surface functionalities (such as epoxide, hydroxyl and carboxylic groups) provides ideal support material for enzyme immobilization [29,30].

Immobilization may also enable the efficient production of biodiesel at an industrial level. There is a great interest in process intensification technologies, which may overcome the major drawbacks of the transesterification reactions [31]; however, due to the technical aspects of enzyme-catalyzed biodiesel synthesis, the development of suitable enzymatic reactors [32] comes up against difficulties. Currently, among the different reactors developed for biodiesel production, the most commonly used are the batch-stirred tank reactor and the packed bed reactor.

Although several studies reported high conversion in fatty acid methyl esters (FAMEs)-type biodiesel (over 90% conversion after 30 h with immobilized *Burkholderia sp*. lipase [33]), 97% conversion after 16 h using lipase from Pacific white shrimp [34] and 94% conversion after 10 h with *Rhizomucor miehei* lipase (RML) covalently immobilized on polyamidoamine dendrimer-coated magnetic multi-walled carbon nanotubes [35]. According to our knowledge, lipase-catalyzed quasi-complete conversion of triglycerides into fatty acid ethyl esters (FAEE) has not yet been reported.

Since lipase B from *Candida antarctica* covalently immobilized on carboxylated single-walled carbon nanotubes (SwCNT_COOH_) proved to be a highly efficient biocatalyst for biodiesel synthesis [36], and Amano lipase from *Pseudomonas fluorescens* (L-AK) shows an unusually high methanol and ethanol resistance, being efficient in biodiesel production [37] even after its immobilization on hydrophobic supports by adsorption [38], the present study aims to develop a novel, highly active and stable biocatalyst for the conversion of sunflower oil into the corresponding FAEE through the covalent immobilization of L-AK on various hydrophobic carbon nanomaterial supports (carboxy and amino-functionalized single-walled carbon nanotubes (SwCNT_COOH_ and SwCNT_NH2_), and reduced graphene oxide (rGO)) of large surface area, derivatized with various linkers with different lengths.

## 2. Results

### 2.1. Covalent Immobilization of L-AK on Nanomaterials

**Scheme 1:**
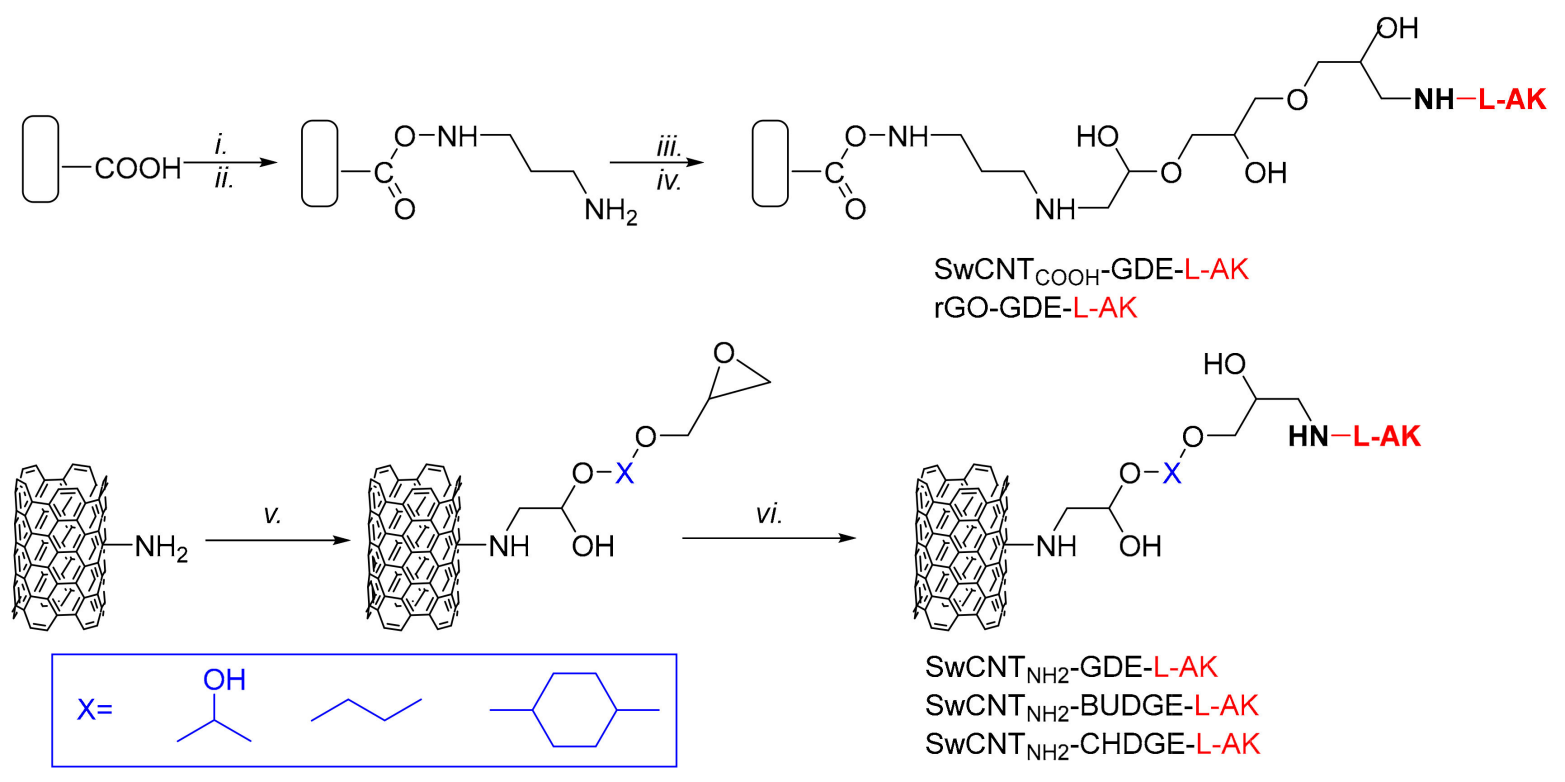
Immobilization of *Pseudomonas fluorescens* (L-AK) on nanosupports: (**i**) CDI in CH_2_Cl_2_; (**ii**) H_2_N-(CH_2_)_3_-NH_2_ in water; (**iii**) glycerol diglycidyl ether (GDE) in CH_2_Cl_2_; (**iv**) L-AK in PBS buffer (20 mM Na_2_HPO_4_, 150 mM NaCl, pH 7); **v**) diglycidyl ether in CH_2_Cl_2_; (**vi**) L-AK in PBS buffer (20 mM Na_2_HPO_4_, 150 mM NaCl, pH 7).

The immobilization of the lipase through covalent binding on nanomaterials was performed by similar approaches (Scheme 1) based on the protocol previously developed by us [35]. The nanosupport (SwCNT_COOH_ or rGO) was activated with *N,N’*-carbonyldiimidazole (CDI, Scheme 1, step *i*), to the CDI-activated support 1,3-propanediamine (Scheme 1, step *ii*) and then glycerol diglycidyl ether (GDE) as crosslinker (Scheme 1, step *iii*) were gradually bonded. The enzyme L-AK was finally reacted with the bisepoxide-activated support (Scheme 1, step *iv*), resulting in two biocatalysts, SwCNT_COOH_-GDE-L-AK and rGO-GDE-L-AK.

Activated SwCNT_NH2_ was obtained in a single step (Scheme 1, step *v*) from SwCNT_NH2_ and GDE and further used for the enzyme covalent attachment using the free amino groups of L-AK, resulting in the desired SwCNT_NH2_-GDE-L-AK, as presented in Scheme 1.

Both immobilization protocols provided high immobilization yields (>99% of the lipase bound to the support) and were carried out in presence of the non-ionic surfactant Tween 80, known for its benefic influence upon the fixation of the enzyme in a more active open conformation, enhancing the activity of the formed biocatalyst [39].

### 2.2. Transesterification Reactions Mediated by Immobilized L-AK

Further, the catalytic properties of the immobilized enzyme preparates were tested in the transesterification of sunflower oil with ethanol. The reaction conditions were optimized in order to achieve the highest conversion rate, focusing on the effect of the nature of organic solvents, water content of the reaction mixture, substrate:nucleophile ratio and the enzyme load. For the preliminary experiments, three biocatalysts, SwCNT_COOH_-GDE-L-AK, SwCNT_NH2_-GDE-L-AK and rGO-GDE-L-AK, with an enzyme load of 0.33 mg enzyme/mg immobilized preparation were used as model biocatalysts.

#### 2.2.1. The Effect of Organic Solvents upon Biodiesel Production

**Figure 1:**
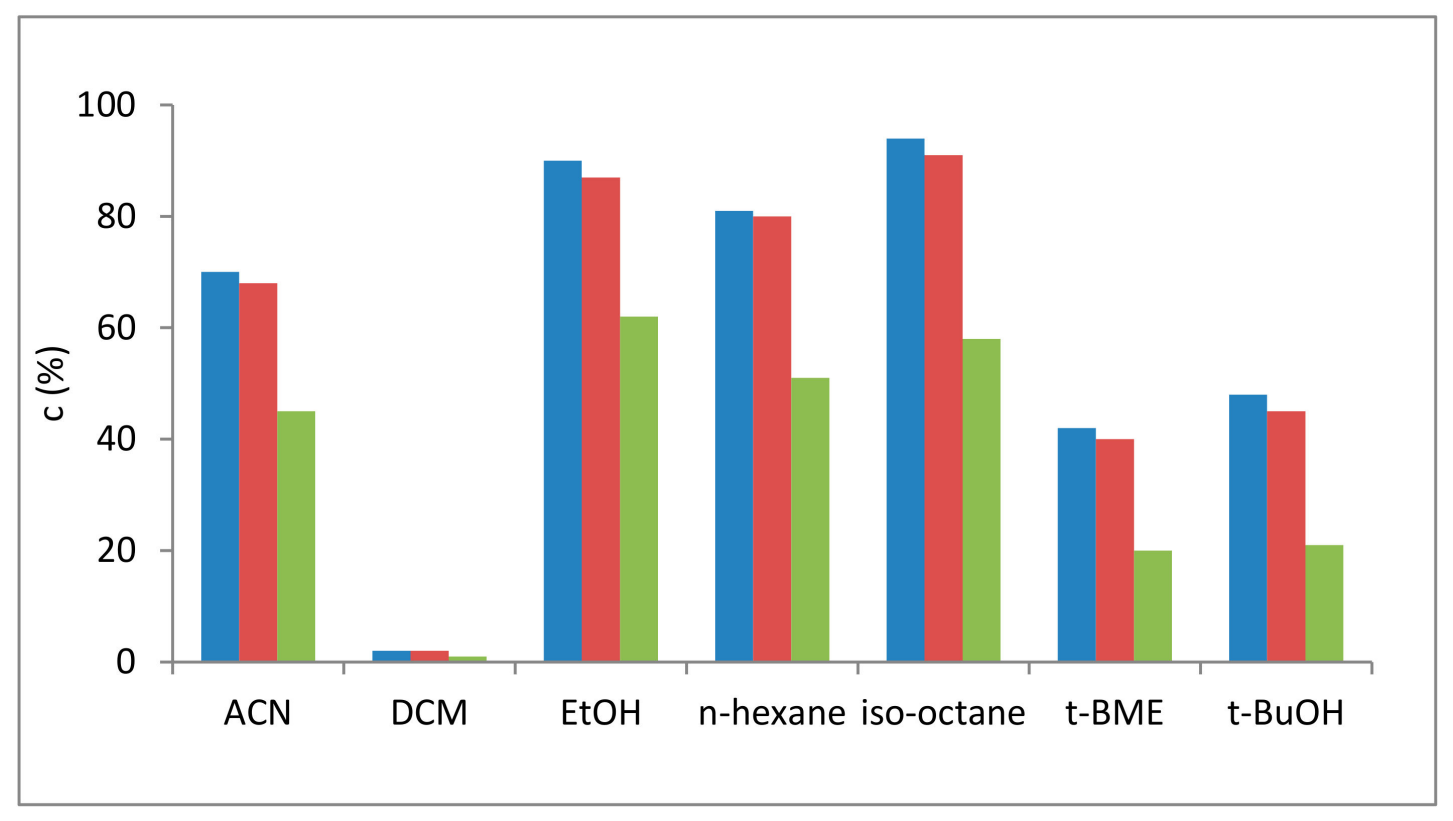
The influence of the solvent on the ethanolysis of sunflower oil catalyzed by single-walled carbon nanotubes (SwCNT)_NH2_-GDE-L-AK (blue bars), SwCNT_COOH_-GDE-L-AK (red bars) and reduced graphene oxide (rGO)-GDE-L-AK (green bars) (4 h).

The enzymatic ethanolysis of the sunflower oil was performed with all enzyme preparates in various solvents at the same enzyme:substrate ratio, identic substrate and ethanol concentrations, all at 30 °C. Samples were taken after 4 h reaction time and the process was monitored with ^1^H-NMR spectrometry. While in dichloromethane the biocatalysts were almost inactive, in *tert*-butanol and in *tert-*butylmethylether (*t*-BME), the L-AK preparates displayed moderate activities. Since in *n*-hexane, neat ethanol and acetonitrile the enzymatic transesterification reactions underwent good conversions, *iso-*octane was selected as the best solvent, allowing the maximal transformation after 4 h with two biocatalysts. While SwCNT_COOH_ and SwCNT_NH2_ were appropriate carriers for L-AK covalent binding (*c* > 90% in *iso-*octane), L-AK immobilized on rGO displayed unfortunately reduced enzyme activity (*c* > 58% in *iso-*octane) for biodiesel production (Figure 1). As a consequence, this biocatalyst was not included in further experiments.

#### 2.2.2. The Effect of Water Content on Biodiesel Production

The activity and selectivity of lipases are strongly influenced by the water content of the reaction mixture. The presence of a certain minimal amount of water is crucial to maintain the conformational mobility of the protein, with direct influence upon the efficacy of the lipase preparate. However, in the case of the alcoholysis of tryglicerides in various organic solvents, the influence of the water content upon the efficacy of various lipases is fuzzy. While the synthesis of fatty acid alkyl esters mediated by *Candida antarctica* lipase B (CALB) is negatively influenced by the increasing of the water content of the reaction mixture [40], the efficacy of lipases from other *Candida* species, from *Rhizopus oryzae* and from *Pseudomonas cepacia* increased with water content of the reaction mixture [41,42].

**Figure 2:**
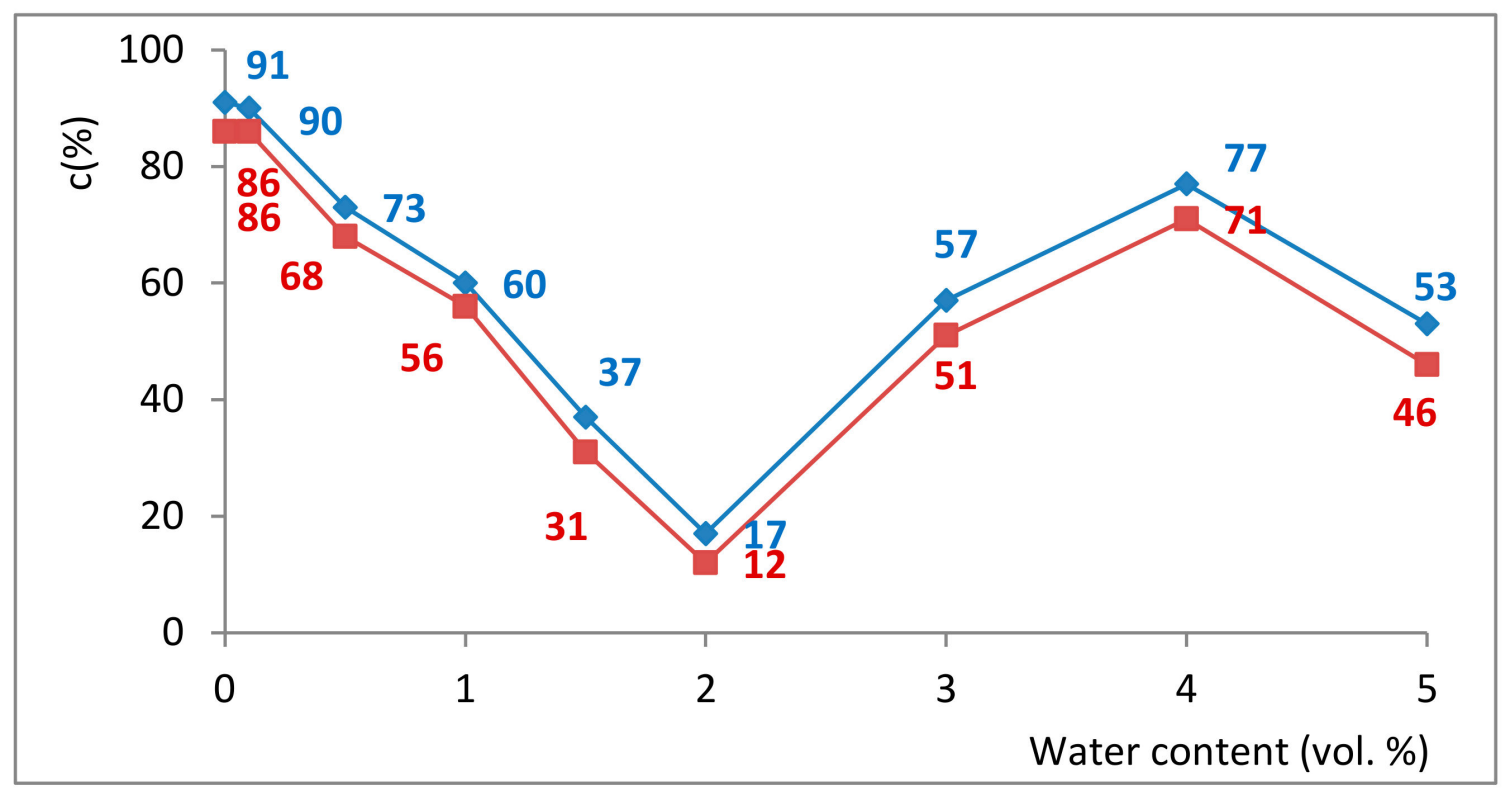
The effect of water content on the biodiesel production with SwCNT_COOH_-GDE-L-AK (red points) and SwCNT_NH2_-GDE-L-AK (blue points) in *iso*-octane (4 h).

In this context, the influence of the water content (0–5%, *v*/*v*) of the reaction mixture upon the SwCNT_COOH_-GDE-L-AK and SwCNT_NH2_-GDE-L-AK catalyzed ethanolysis of sunflower oil was next investigated (Figure 2). Increasing the water concentration from 0.1 to 2% *v*/*v*, a linear drastic decrease of the conversion into biodiesel was observed. Interestingly, further increase of the water concentration gradually enhanced the enzyme activity with a local maximum at 4% *v*/*v* water content.

Probably, in the presence of water, the hydrolysis reaction competes with biodiesel formation, and as shown in Figure 2, the highest enzyme activity was achieved in the absence of water.

#### 2.2.3. The Influence of the Protein Content of the Biocatalysts upon the Specific Enzyme Activity for the Transesterification Reactions

**Figure 3:**
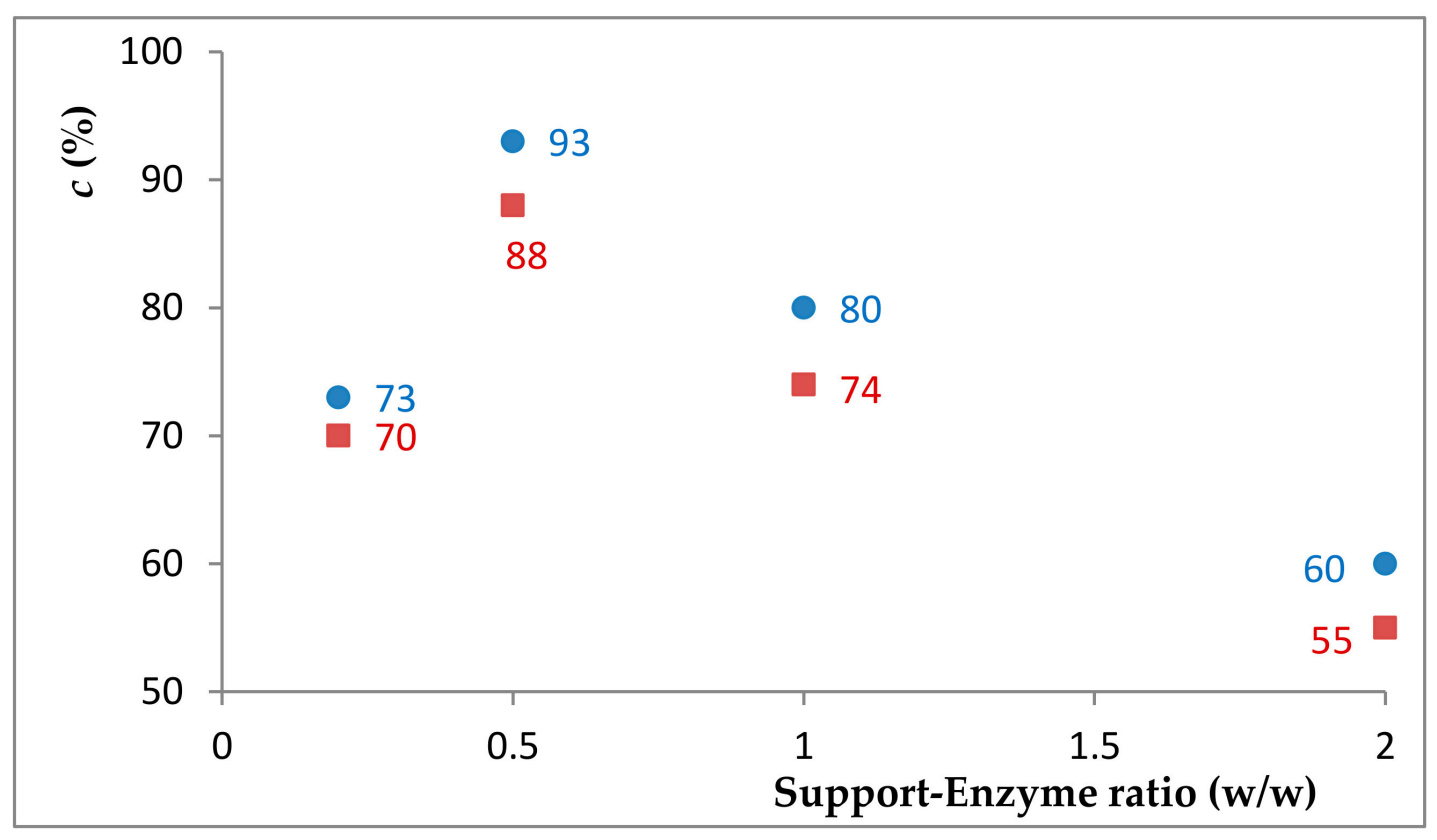
The effect of the enzyme load on the conversion of the transesterification reaction (3 h) with SwCNT_COOH_-GDE-L-AK (red points) and SwCNT_NH2_-GDE-L-AK (blue points).

Besides the used immobilization method and the nature of the support, the catalytic performance of the biocatalyst could also be influenced by the enzyme load of the biocatalyst. The large surface area and the presence of ~3–5% *w*/*w* content of the active carboxy or amino functional groups of the tested SwCNT_COOH_ and SwCNT_NH2_ carbon nanomaterials enabled the covalent binding of even 4 mg protein/1 mg support, [43] which could lead to the partial inactivation of enzymes caused by the steric hindrance of the protein molecules. Since, until this stage, preparates with an enzyme load of 0.33 mg L-AK/mg biocatalyst were used, the ethanolysis of sunflower oil was further tested in the presence of biocatalysts with various protein content (Figure 3). The highest activity (~93% conversion after 3 h) was obtained for the biocatalyst containing around 66% L-AK (corresponding to a support:enzyme ratio of 1:2, *w*/*w*).

#### 2.2.4. The Effect of the Oil-Ethanol Molar Ratio

Since the amount of nucleophile can significantly influence the reaction rate of the biocatalytic transesterification processes, the biocatalyst’s activity at various oil:ethanol ratios was tested (Table 1). Using low, stoichiometric ethanol content, including the 1:5 oil:ethanol ratio of the previous experiments, the activity of both biocatalysts was high, achieving maximal conversions (97–98% conversion in 3 h, Table 1, Entry 5) at a 1:7 oil:ethanol molar ratio. Higher ethanol content caused the continuous decrease of the conversions probably due to the presence of insoluble alcohol droplets in oil that caused denaturation of the enzyme, as already reported for enzymatic methanolysis of oils [44].

#### 2.2.5. Reusability of the Immobilized Lipases

As demonstrated recently by Hessel [45], a major cost for the scaled-up production of biodiesel in a continuous flow reactor is related to the enzyme costs influenced by the support material itself. However, when the immobilization yield and the biocatalyst stability are high, the enzymatic process is in general economically viable, sustainable and environmentally friendly, as compared with the chemical classic routes.

**Figure 4:**
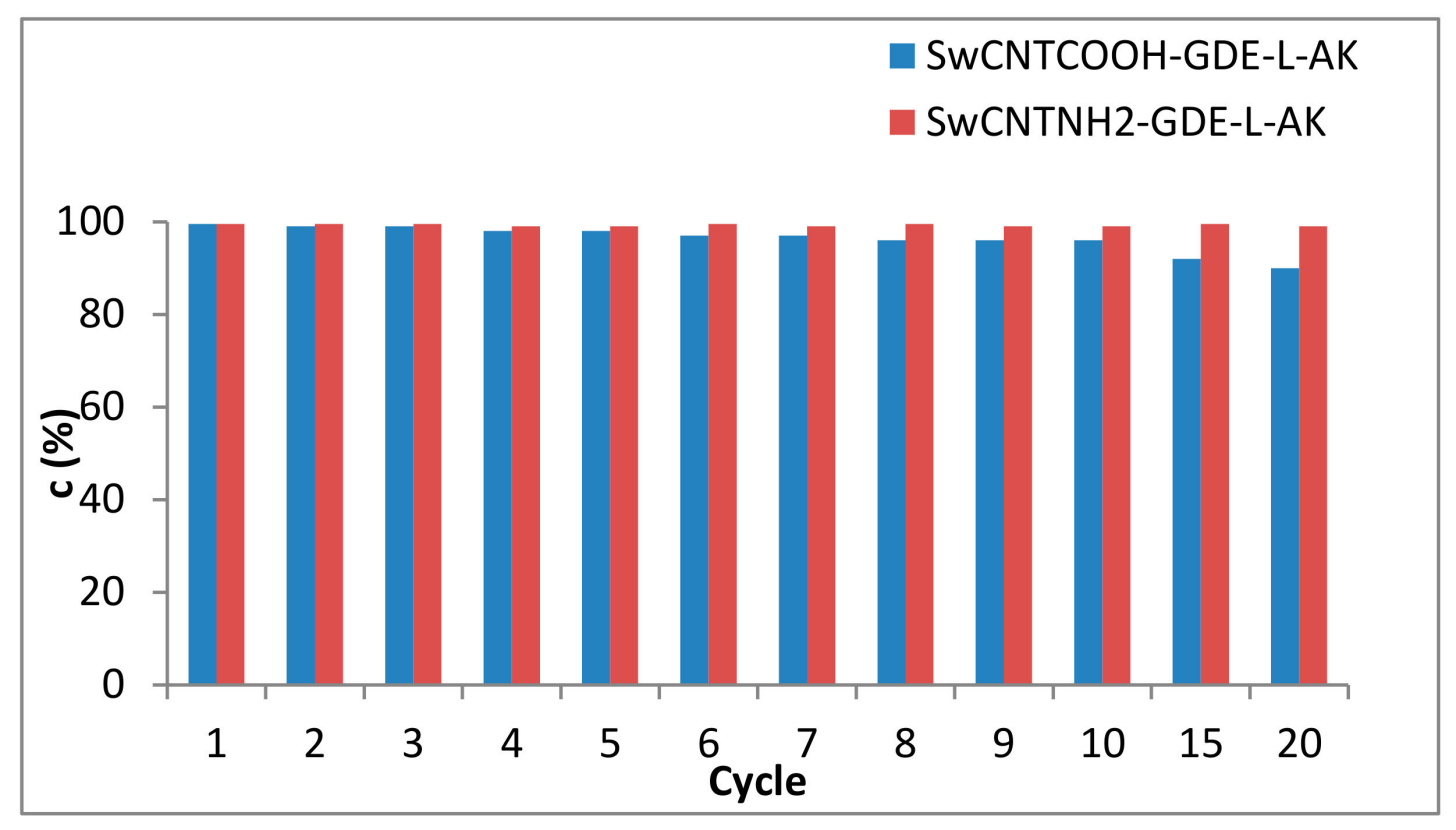
The time profile of the SwCNT_NH2_-GDE-L-AK (red bars) and SwCNT_COOH_-GDE-L-AK (blue bars) mediated transesterification of sunflower oil after 4 h (dry *iso*-octane, oil-ethanol 1:5 molar *ratio*).

The covalently attached lipase on the support material should be suitable for long-term use since the leakage of the enzyme from the nanotubes is disabled. Further, the reusability of SwCNT_COOH_-GDE-L-AK and SwCNT_NH2_-GDE-L-AK (0.66 mg L-AK/mg preparate) was tested in repeated batch cycles (Figure 4).

Both enzyme preparates presented high activity. While for SwCNT_COOH_-GDE-L-AK, a small and gradual inactivation was measured, the near-complete transformation of the sunflower oil (>99% conversion) was observed even after 20 reaction cycles when SwCNT_NH2_-GDE-L-AK was used, this preparate providing an excellent operational stability since only a small activity decrease (approx. 0.5%) was detected even after 20 reaction cycles.

#### 2.2.6. Other Linkers for Enzyme Immobilization on SwCNT_NH2_

Finally, in our attempt to increase the activity of the lipase preparation, besides GDE, other cross-linkers with different lengths and structures were used as spacer arms for the L-AK covalent immobilization on SwCNT_NH2_. For this scope, 1,4-butanediol diglycidyl ether and 1,4-cyclohexanedimethanol diglycidyl ether were used for immobilization (Scheme 1) and the newly prepared bionanoconjugates (SwCNT_NH2_-BuDGE–L-AK and SwCNT_NH2_-CHDGE–L-AK) were further tested in the previously found optimal conditions for oil ethanolysis in several cycles.

**Figure 5:**
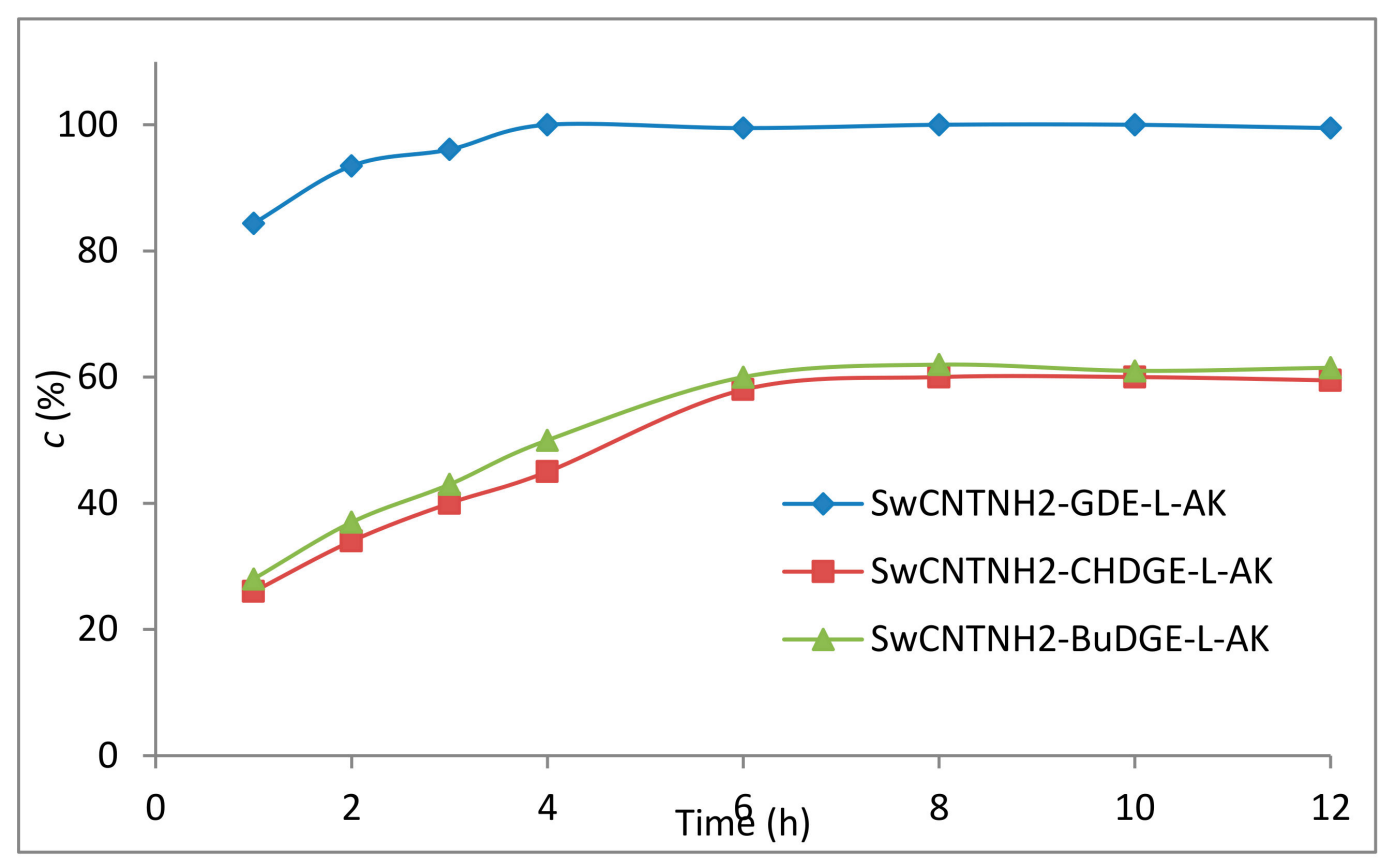
The time profile of the immobilized L-AK-mediated transesterification of sunflower oil.

The conversions obtained using the SwCNT_NH2_-CHDGE–L-AK with the more rigid cyclohexane-type linker, or the SwCNT_NH2_-BuDGE–L-AK with a more mobile but hydrophobic butyl spacer arm were moderate compared to those obtained with SwCNT_NH2_-GDE-L-AK (Figure 5). Moreover, inspecting the time course of the SwCNT_NH2_-BuDGE–L-AK and SwCNT_NH2_-CHDGE–L-AK-catalyzed reactions, in both cases, the productivities are unsatisfactory, with only ~60% maximal conversion of oil into the desired FAEE being observed.

## 3. Materials and Methods

### 3.1. Materials

Native Amano lipase from *Pseudomonas fluorescens* (L-AK) was purchased as lyophilized powder from Chiral Vision, Netherlands. Graphene, single-walled carbon nanotubes (SwCNT; ID = 0.8–1.6 nm, OD 1–2 nm, length = 5–30 µm) and carboxylated single-walled carbon nanotubes (SwCNT_COOH_) (COOH content: 2.73 wt%; ID = 0.8–1.6 nm, OD = 1–2 nm, length = 5–33 µm) were purchased from Chengdu Organic Chemicals Co. Ltd., China. *N*,*N*’-Carbonyldiimidazole and 1,3-propanediamine were products of Alfa Aesar (Ward Hill, MA, USA). Glycerol diglycidyl ether (GDE), 1,4-butanediol diglycidyl ether, 1,4-cyclohexanedimethanol diglycidyl ether, Bradford reagent for protein determination, Tween-80 and C*D*Cl_3_ were purchased from Sigma-Aldrich (St. Luis, MO, USA). The sunflower oil was obtained from a local store. All solvents, technical grade, were dried and/or freshly distilled prior to use.

### 3.2. Equipments

For the Bradford protein assay, an Agilent 8453 UV–Vis spectrophotometer was used. During enzyme immobilization, ultrasonications were performed in a Transsonic 460/H ultrasonic bath, Elma Schmidbauer GmbH, at 100 W and 40 kHz.

The shaking and incubation of enzymatic reactions were performed using a Titramax 1000 instrument, equipped with a heating module. ^1^H-NMR spectra were recorded in C*D*Cl_3_ as solvent at 25 °C on Bruker Avance 400 and Bruker Avance 600 NMR spectrometers, operating at 400 and 600 MHz, respectively. The ^1^H-NMR spectral parameters were: spectral width 12,019.2 Hz, acquisition time 2.99 s and number of scans 32. Signals were expressed in ppm on the δ scale.

GC analysis was performed on an Agilent 7890A GC gas chromatograph equipped with a flame ionization detector on a DB-WAX capillary column (30m × 0.32mm × 0.5μm).

Elemental analyses were carried out with a Vario Micro Cube analyzer, Elementer Analysen Systeme GmBH (Langeselbold, Germany).

The amination reaction was carried out at 600 W power at 50 bar pressure at 300 °C for 1 h in a CEM microwave-assisted reactor (CEM Corporation (Matthews, NC, USA)).

All mixtures containing SwCNTs were filtered on a PTFE membrane filter with 0.22 µm pore size, Membrane-solutions, Nantong Co., Ltd. (Shanghai, China).

### 3.3. Methods

#### 3.3.1. Determination of Conversion

The conversions of the transesterification reactions were determined as earlier described [35] recording the ^1^H-NMR spectra of the reaction mixtures. Reference spectra of the authentic biodiesel, sunflower oil and their mixtures were used for calibration (see Supplementary Materials).

#### 3.3.2. Functionalization of SwCNT with Amino Groups

The functionalization of carbon nanotubes was carried out as earlier described [46]. SwCNT (400 mg) and urea (400 mg) were suspended in dimethyl formamide (DMF, 5 mL). The reaction was carried out in a microwave-assisted reactor at 300 °C for 1h. The functionalized SwCNT was washed with DMF, methanol, CH_2_Cl_2_ and dried. The obtained aminated support SwCNT_NH2_ contained ~4.8% NH_2_ (determined by elemental analysis).

#### 3.3.3. Fatty Acid Composition of Sunflower Oil

The fatty acid composition of the sunflower oil was determined according to the AOAC reference procedure as earlier reported using chromatographically pure methyl ester standards [35] (see Supplementary Materials, Figure S1).

#### 3.3.4. Covalent Binding of L-AK to SwCNT_COOH_

Lipase immobilization on carboxylated single-walled carbon nanotubes SwCNT_COOH_ was achieved as previously described (Scheme 1) [35]. In all cases, high immobilization yields characterized the resulted products (>99% of the enzyme bound to the support; enzyme load: 0.33/0.5/0.66/0.83 mg protein/mg immobilized preparation).

#### 3.3.5. Covalent Binding of L-AK to SwCNT_NH2_

SwCNT_NH2_ (20 mg) was incubated with a solution of diglycidyl ether (glycerol diglycidyl ether, 1,4-butanediol diglycidyl ether or 1,4-cyclohexanedimethanol diglycidyl ether, 0.2 mmol) in CH_2_Cl_2_ (5 mL) under shaking (at 1350 rpm) at room temperature overnight, with occasional sonication to avoid bundled SwCNT formation (Scheme 1, step *v*). The mixture was filtered on a membrane filter and then washed with CH_2_Cl_2_ (3 × 1 mL). In the solution of L-AK (40 mg) and Tween-80 (6.5 μL) in 6 mL PBS buffer (20 mM Na_2_HPO_4_, 150 mM NaCl, pH 7, 6 mL), the bisepoxide-activated SwCNT_NH2_ (20 mg) was added and the mixture was shaken at room temperature at 1350 rpm overnight (Scheme 1, step vi). The resulted biocatalyst (SwCNT_NH2_-GDE-L-AK, SwCNT_NH2_-BuDGE-L-AK, SwCNT_NH2_-CHDGE-L-AK) was filtered off on a membrane filter, sonicated several times and washed with water (3 × 10 mL) until no protein trace was detected in the filtrate. After that, immobilized enzymes were freeze-dried and used in enzymatic tests. The amount of the immobilized L-AK on the bisepoxide-activated SwCNT_NH2_ was determined as the difference between the total mass of the enzyme in the solution before immobilization and in the unified filtrates after immobilization. In all cases, high immobilization yields characterized the resulted products (>99% of the enzyme bound to the support; enzyme loading: 0.66 mg protein/mg immobilized preparation). For different enzyme loading, the corresponding L-AK quantities were used.

#### 3.3.6. Covalent Binding of L-AK to Reduced Graphene Oxide (rGO)

Reduced graphene oxide (rGO, 20 mg) was activated with *N,N’*-carbonyldiimidazole (CDI, 32.4 mg, 0.2 mmol) in CH_2_Cl_2_ (5 mL) under shaking (at 1350 rpm and room temperature, overnight), with occasional sonication, to avoid bundled rGO formation. After CDI activation, the sample was filtered on a membrane filter and then washed with CH_2_Cl_2_ (3 × 5 mL). Into the suspension of the CDI-activated rGO (20 mg) in distilled water (5 mL), 1,3-propanediamine (0.12 mmol) was added and the reaction mixture was shaken (at 1350 rpm and room temperature, overnight), with occasional sonication to avoid bundled nanoparticle formation. The reaction mixture was filtered on a membrane filter, washed with distilled water (3 × 5 mL) and then dried. In the solution of glycerol diglycidyl ether (GDE, 100 μL) in CH_2_Cl_2_ (5 mL), diamine-coupled rGO (20 mg) was suspended and the reaction mixture was shaken (at 1350 rpm and room temperature, overnight), with occasional sonication. The mixture was filtered on a membrane filter and then washed with CH_2_Cl_2_ (3 × 5 mL). In the solution of L-AK (10 mg) and Tween-80 (6.5 μL) in 6 mL PBS buffer (20 mM Na_2_HPO_4_, 150 mM NaCl, pH 7), the bisepoxide-activated rGO (20 mg) was added and the mixture was shaken at room temperature at 1350 rpm, overnight. The resulted biocatalyst (rGO - GDE-L-AK) was filtered off on a membrane filter and, sonicated several times and washed with water (3 × 10 mL) until no protein trace was detected in the filtrate. After that, the immobilized enzyme was freeze-dried and used in enzymatic transesterifications. The amount of the immobilized L-AK on the bisepoxide-activated rGO was determined as the difference between the total mass of the enzyme in the solution before immobilization and in the unified filtrates after immobilization (>99% of the enzyme bound to the support; enzyme loading: 0.33 mg protein/mg biocatalyst).

#### 3.3.7. Biodiesel Production by Enzymatic Ethanolysis

*General procedure*: reactions were performed using 20 mg sunflower oil (approx. 23 µmol) in 500 μL organic solvent, 3 mg enzyme preparate and 6.5 µL ethanol (approx. 110 µmol). The reaction mixtures were shaken (1350 rpm) at room temperature for 3–4 h. The mixtures were centrifuged at 9840× *g* (13,400 rpm) for 1 min, the solvent was removed from the supernatant at reduced pressure and the obtained biodiesel was analyzed using ^1^H-NMR spectroscopy.

In order to determine the optimal solvent for the transesterification reaction, several organic solvents were tested in the general procedure (acetonitrile, dichlorometane, ethanol, *n*-hexane, *iso*-octane, methyl tert-butyl ether and *tert*-butanol). In 500 μL organic solvent, 20 mg sunflower oil, 3 mg enzyme preparate and 6.5 µL ethanol were added. The reaction mixtures were shaken (1350 rpm) at room temperature for 4 h. The mixtures were centrifuged at 9840× *g* (13,400 rpm) for 1 min, the solvent was removed from the supernatant at reduced pressure and the obtained biodiesel was analyzed using ^1^H-NMR spectroscopy.

Different amounts of water were added to the *iso-*octane used as a solvent for the enzymatic transesterification reaction with the aim to find the optimal water content. In 500 μL iso-octane (containing 0–5%, *v*/*v* water), 20 mg sunflower oil, 3 mg enzyme preparate and 6.5 µL ethanol were added. The reaction mixtures were shaken (1350 rpm) at room temperature for 4 h. The mixtures were centrifuged at 9840× *g* (13,400 rpm) for 1 min, the solvent was removed from the supernatant at reduced pressure and the obtained biodiesel was analyzed using ^1^H-NMR spectroscopy.

The effect of the enzyme load was studied using biocatalysts with different support:enzyme ratios. In 500 μL *iso*-octane, 20 mg sunflower oil, the corresponding amount of the enzyme preparate and 6.5 µL ethanol were added. The reaction mixtures were shaken (1350 rpm) at room temperature for 3 h. The mixtures were centrifuged at 9840× *g* (13,400 rpm) for 1 min, the solvent was removed from the supernatant at reduced pressure and the obtained biodiesel was analyzed using ^1^H-NMR spectroscopy.

During the optimization of the oil:ethanol molar ratio, different oil:ethanol mixtures were used. In 500 μL *iso*-octane, 20 mg sunflower oil, 1.5 mg enzyme preparate and the corresponding amount of ethanol (1:1, 1:3, 1:5, 1:6, 1:7, 1:8, 1:10, 1:20, 1:50 oil:ethanol molar ratio) were added. The reaction mixtures were shaken (1350 rpm) at room temperature for 3 h. The mixtures were centrifuged at 9840× *g* (13,400 rpm) for 1 min, the solvent was removed from the supernatant at reduced pressure and the obtained biodiesel was analyzed using ^1^H-NMR spectroscopy.

*Optimized procedure*: The mixture of 20 mg sunflower oil in 500 μL *iso*-octane, 1.5 mg biocatalyst containing 1 mg L-AK and 6.5 µL ethanol was shaken (1350 rpm) at 30 °C for 3 h. The mixture was centrifuged at 9840× *g* for 1 min (13,400 rpm), the solvent was removed from the supernatant at reduced pressure and the obtained biodiesel was analyzed using ^1^H-NMR spectroscopy.

## 4. Conclusions

In the present study, Amano lipase from *Pseudomonas fluorescens* (L-AK) has been covalently immobilized on functionalized single-walled carbon nanotubes and reduced graphene oxide and the obtained enzyme preparations were used for the production of biodiesel in the batch system. The most active lipase preparate was achieved when amino-functionalized carbon nanotubes as support and GDE as crosslinker (SwCNT_NH2_-GDE-L-AK) were used. Under the optimal conditions of the batch procedure, the novel biocatalyst provided high activity. With almost complete conversions of the sunflower oil to biodiesel in only 4 h and impressive stability, the activity was maintained unaltered even after 20 reaction cycles.

## Figures and Tables

**Table 1:** The effect of oil:ethanol molar ratio on the ethanolysis process catalyzed by SwCNT immobilized L-AK (3 h reaction time).

Entry	Oil:Ethanol Molar Ratio	Conversion (%)	
		SwCNT_COOH_-GDE-L-AK	SwCNT_NH2_-GDE-L-AK
1	1:1	67.1	68.8
2	1:3	93.1	94.2
3	1:5	96.1	96.8
4	1:6	96.8	97.4
**5**	**1:7**	**97.1**	**98.0**
6	1:8	94.1	95.2
7	1:10	93.7	94.5
8	1:20	94.9	95.2
9	1:50	67.1	68.9

## Supplementary Materials

The following are available online. Determination of conversion values through ^1^H-NMR; 2. Fatty acid composition of sunflower oil; 3. Biodiesel production through basic ethanolysis.
