# Supplementary material for: Efficient Biodiesel Production Catalyzed by Nanobioconjugate of Lipase from Pseudomonas fluorescens
Source: Molecules. 2020 Feb 3;25(3):651. doi: 10.3390/molecules25030651 (PMC7037990; doi:10.3390/molecules25030651)
Supplement: Supplementary file 1 [file molecules-25-00651-s001.pdf]

Efficient biodiesel production catalyzed by nanobioconjugate of lipase from *Pseudomonas fluorescens*

Judith-Hajnal Bartha-Vári[1], Mădălina Elena Moisă[1], László Csaba Bencze[1], Florin Dan Irimie[1], Csaba Paizs, Monica Ioana Toșa[1]*

[1] Babeș-Bolyai University, Biocatalysis and Biotransformation Research Center, Arany János 11, Cluj-Napoca, 400028-România; chem@chem.ubbcluj.ro

* Correspondence: mtosa@chem.ubbcluj.ro; Tel.: +40-264-593833

Supplementary materials

1. Determination of conversion values through ^{1}H-NMR

The ^{1}H-NMR spectra of the authentic biodiesel, sunflower oil and their mixture was recorded. Since the quartet signal generated by the protons associated with the methylene group belonging to the ethyl moiety of the fatty acids ethyl esters of the biodiesel and doublet of doublet signals associated with the methylene protons belonging to glyceryl moiety of the oil are overlapping a calibration curve was determined in order to calculate the reaction conversions [1].

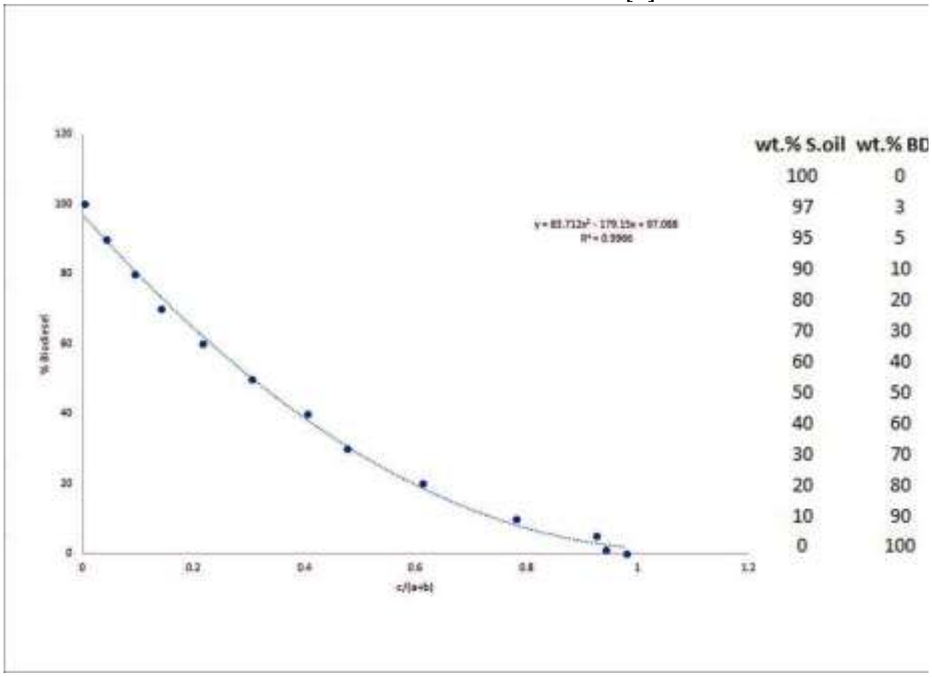

Figure S1. The calibration curve used to determine the conversion of the transesterification reactions

From the known biodiesel content calculated from the calibration curve, the conversion of the transesterification reaction can be calculated using the equations described below:

$$c = \frac{n_{oil\ transformed}}{n_{oil\ total}} \times 100 \qquad \text{(equation 1)}$$

$$n_{oil\ transformed} = \frac{1}{3} \times n_{BD} \qquad \text{(equation 2)}$$

$$n_{oil\ total} = n_{oil\ transformed} + n_{oil\ remained} \quad \text{(equation 3)}$$

Based on these equations, the conversion can be calculated with the formula:

$$c = \frac{1}{1 + 3 \times \frac{100 - BD_{content} \times M_{\mathrm{BD}}}{BD_{content} \times M_{Oil}}} \times 100$$

2. Fatty acid composition of sunflower oil [1]

Fatty acid	Palmitic acid	Stearic acid	Oleic Acid	Linoleic acid	γ-Linolenic acid	Docosanoic acid
**Abbreviation**	C16:0	C18:0	C18:1 (9)	C18:2 (9,12)	C18:3 (6,9,12)	C22:0
**Content (% w/w)**	6.5	3.6	26.7	62.3	0.5	0.4

3. Biodiesel production through basic ethanolysis

The basic ethanolysis was achieved as earlier described [1] (In a 100 mL two-necked flask, equipped with mechanical stirrer and condenser, the mixture of sunflower oil (50 mL), ethanol (25 mL) and NaOH (0.5 g) as catalyst was stirred and heated to reflux in a silicone oil bath for 3 hours. The reaction was monitored by TLC (petroleum ether-ethyl acetate 9:1, v/v, visualization by iodine vapours). After the completion of the reaction, the mixture was allowed to cool to room temperature and the excess of ethanol was removed on a rotary evaporator at reduced pressure. The obtained crude reaction mixture was partitioned in petroleum ether (20 mL) and water (20 mL) adjusting the pH to 7 with phosphoric acid. The isolated organic layer was washed with water (3×20 mL), then dried over anhydrous magnesium sulfate (2 g). Petroleum ether was removed on a rotary evaporator, yielding a crude, yellowish oil, from which biodiesel was obtained as colorless oil through vacuum distillation (b.p. 221-222 °C/ 16-17 Hg mm).

4. References

1. Bencze, L.C., Bartha-Vári, J.H., Katona, G., Toșa, M.I., Paizs, C., Irimie, F.D. Nanobioconjugates of *Candida antarctica* lipase B and single-walled carbon nanotubes in biodiesel production. *Bioresource Technol.* **2016**, 200, 853–860.
